# Sustainable transportation: Definitions, dimensions, and indicators – Case study of importance-performance analysis for the city of Tehran

**DOI:** 10.1016/j.heliyon.2023.e20457

**Published:** 2023-10-05

**Authors:** Isa Heidari, Abbas Toloie Eshlaghy, Seyyed Mohammad Seyyed Hoseini

**Affiliations:** a Faculty of Management and Economics, Industrial Management Department, I.A.U. Science and Research Branch, Tehran, Iran; b Faculty of Industrial Engineering, Industrial Engineering Department, Iran University of Science and Technology, Tehran, Iran

**Keywords:** Sustainable transportation, Environmental indicators, Social indicators, Economic indicators, Importance-performance analysis

## Abstract

Despite the advantages of transportation development, its negative consequences, including greenhouse gas (GHG) emissions, air pollution, increasing energy consumption, traffic, and accidents in transportation, have caused serious concern in the world community. Following the global efforts to introduce the framework for sustainable development (SD), the sustainability concept entered the transportation of literature known as sustainable transportation (ST). We didn't find a universally agreed definition and indicators for (ST). To overcome this limitation, we extracted special terms in different definitions of (ST) and widely used indicators in selected articles. Using 60 widely used indicators and the importance-performance analysis (IPA) method, we evaluated the transportation of Tehran. Like some metropolises, Tehran city has high air and noise pollution, congestion, traffic, and accidents. The main advantage of our research is the possibility to simultaneously assess the importance, performance, and prioritize performance of indicators for improvement, with optimal cost and time. Our evaluation showed that 41 indicators (68.34%) have high importance, but their performance is low and should be improved. 80% of these indicators were related to social and environmental dimensions. We prioritized Tehran's transportation indicators and provided recommendations to improve their performance. Prioritizing indicators showed that improving the performance of Tehran's transportation, reducing natural resource consumption, attention to human health, and reducing energy consumption have high priority. Finally, we have provided a comprehensive definition (ST) of widely used terms.

## 1 Introduction

Always transportation, has been one of the basic needs in the life of human societies. However, its expansion in recent years has faced principal challenges. Transportation accounts for approximately 25% of carbon dioxide emissions worldwide [1]. In transportation, energy consumption, carbon dioxide emissions, and other pollutants are increasing faster than in any other sector [2]. In transportation, energy consumption, carbon dioxide emissions, and other pollutants are increasing faster than in any other sector. The population is more than 7.76 billion in the world now. The expected to reach more than 9.7 billion people by 2050 [1]. The population growth and the intensification of the urbanization process started in the middle of the 20th century and continued faster in the current century [3]. About 70% of the world's population will live in urban areas by 2050 [4]. Urbanization has increased trips, the movement of goods, and the development of communication and transportation. In metropolitan cities in developed and developing countries, transportation problems have reached critical dimensions. The physical and mental health of the urban population is seriously affected by air pollution, noise pollution, accidents, congestion, and traffic [5].

The range of challenges between the importance of transportation development and its negative consequences is getting wider every day. However, it is almost impossible to determine the base of countering the harmful effects of transportation development. Brundtland's report (1987) is the basis of the world community's action to find a way to control the negative consequences of providing the needs of the present generation. Also, this report emphasizes the preservation of interests and the ability to supply the needs of future generations. This report aims to achieve balance in the environmental, social, and economic aspects of development, known as sustainable development (SD). Over time, the definition of (SD) expanded and included transportation, referred to as (ST). Although it has been emphasized on the simultaneous attention to three environmental, social, and economic dimensions, and until now, many definitions of (ST) and its indicators have presented, there isn't agreement on a specified definition or indicators. This study shows that different and widely indicators have provided for each of these dimensions that may not be applicable in all countries. Based on this, providing a comprehensive definition of (ST) and categorizing the widely used and effective indicators in improving the three dimensions of (ST) seems necessary. It is possible to evaluate the adherence of any country to the definition of (ST) by using widely used indicators and measuring their performance.

The goals of this research are:

A Providing a comprehensive definition for (ST) and categorizing the widely used indicators of its dimensions.
B Evaluating the impact of these indicators with a valid scientific method in a real transportation case.

To achieve the research goals, we designed two multi-step algorithms. During the research process, we identified 2520 articles by searching four websites and finally selected 85 related articles through several filtering steps. Then, we extracted definitions (ST) and widely used indicators (60 indicators) from the selected articles. We evaluated the importance and performance of indicators using the (IPA) method. (IPA) method provides the possibility of evaluating the current status of indicators by simultaneously evaluation and prioritizing their importance and performance at a suitable time and a low cost. We chose the transportation of the Tehran metropolis as a case study in an operational environment. This city has more than nine million people. In Tehran city, air pollution, congestion, traffic, accidents, and other negative transportation consequences of daily are evident. Tehran transportation evaluated using 60 widely used indicators, the opinion of experts, and the (IPA) method. Indicators were prioritized by using the results to improve performance.

The results of (IPA) revealed that the indicators of “Minimizing the consumption of natural resources”, “Attention to human health in transportation policies and programs” and “Reducing energy consumption” in three dimensions are the top priorities for improving performance.

Our evaluation of Tehran transportation with sixty indicators showed that 68.34% of the total indicators have high importance, but their performance is low and should be improved. 80.49% of these indicators are related to environmental and social dimensions. Only 19.51% of these indicators are economic indicators, and the performance of these indicators, must be improved according to experts. This high percentage shows the level of concern among experts regarding the potential environmental and social consequences of transportation in Tehran. Only 8.33% of indicators had high importance and performance. 20% had low importance and low performance. 3.33% of indicators had low importance and high performance. Finally, with widely used terms inside definitions, we introduced our summary as a definition for (ST) and then presented our recommendations to improve the performance of indicators in Tehran transportation.

## 2 Literature review

### 2.1 Sustainable transportation: definitions

Since the middle of the 20th century, at the same time as the industrial and economic development of different societies has accelerated, the world population has also grown significantly. This increasing trend has expanded the demand for travel and the movement of goods. Economic development and expansion of transportation, excessive consumption of natural resources, and the resulting pollution have caused serious concerns in the world community. Creating these concerns in the world community and trying to improve this practice led to the emergence of the concept of (SD) [6]. In other words, (SD) is the outcome of increasing people's concern about environmental quality, social and economic vitality, and the threat of global climate change [7].

Economic development has not considered environmental, intergenerational, and livability effects. Most businesses have not been responsible for the costs imposed on society and the Environmental damage and related reconstruction costs [6]. Although, no commonly accepted definition of sustainability about (SD) or (ST) is available [8]. However, it's generally accepted that (SD), and in particular, sustainable transport, requires Finding the right balance between the conditions (present and future) in environmental, social and economic dimensions [8].

The term (SD), in modern literature, was proposed by the special committee of the United Nations (UN) general assembly for long-term environmental strategies to achieve (SD) until the year 2000 and the years after that [9]. This committee later became known as the Brundtland commission. According to this commission, (SD) is the development that meets the needs of the present without compromising the ability of future generations to meet their needs [9,10,11]. Also, Brundtland's report has declared that (SD) creates an optimal balance between economic, environmental, and social dimensions of sustainability [10]. Today, (SD) is a well-known term that shows the need to integrate the economic, social, and environmental aspects of development and politics [12]. In this regard, (World Bank, 1996) defines sustainability more broadly, including environmental, social, and economic dimensions. According to this definition, environmental sustainability preserves natural resources, minimizes pollutants, and reduces impacts on ecosystems as climate change. Social sustainability considers health and safety considerations, access, and distribution of benefits and costs among community groups, and economic sustainability focuses on economic growth, cost-effectiveness, and financial sustainability [13].

In the past two decades, (ST) has become a fundamental goal in transportation planning and policy. At the highest level, (ST) can be called (SD) in the transportation sector [7]. Extensive research has been done on defining and determining the conditions for (SD), but relative research on (ST) is few [7]. Although there is a single and collective definition of (ST), the Canadian Department of Transportation believes that all transportation activities must be sustainable from three aspects: economic, environmental, and social [7]. The main focus of (ST) has been on reducing resource consumption and controlling the environmental degradation and pollution caused by the consumption of petroleum derivatives in cars, and it is the result of people's widespread concern about global warming, which is part of (SD) [7]. Most authors believe that “(ST)” comes from the idea of (SD) [7]. In a broad definition, (ST) considers economic and social well-being, equity, human health, and environmental integrity [14].

The (ST) center has defined (ST) as follows: A (ST) system meets the following criteria: the emission of pollution and waste within the planet's ability to absorb them is limited. It minimizes the consumption of renewable resources to a level of sustainable performance, reuses and recycles its components, reduces land use and noise generation, and allows the basic needs of individuals and communities to be accessed safely and in a manner consistent, with human and ecosystem health, and equality within and between generations be fulfilled. It is cost-effective and efficient, allows the choice of different modes of transportation, and supports a vibrant economy [10,15] (10.13039/501100004347ST). significantly contributes, to a wide range of environmental problems, including energy [7]. Different elements (for example: economic, social, and environmental) of (ST) should be pursued separately [7]. A (ST) system should be safe, efficient, and environmentally friendly [7]. The goal of (ST) is to ensure: that environmental, social, and economic considerations are included in decisions affecting transportation activity [6]. A (ST) system is one in which fuel consumption, vehicle emissions, safety, congestion, and social and economic accessibility are such that they can continue into the indefinite future without causing vital or irreversible damage [6].

(ST) provides the needs of the present without creating problems for future transportation systems by:

1) The use of renewable resources does not exceed their regeneration rate.
2) The use of non-renewable resources does not exceed the rate of development of sustainable renewable alternatives.
3) The emission of pollution does not exceed the absorption capacity of the environment [16].

(ST) should ensure neat and clean streets, preserve the environment and support a dynamic economy [16] (ST). has two definitions. The former focuses on environmental problems and resource depletion. While the latter includes social and economic well-being [16]. (ST) encourages people to seek integrated solutions [16]. (ST) uses energy efficiently [16]. The transportation system must guarantee safety, social interaction, and availability [16]. Any sustainable system should be efficient in energy consumption and create minimal waste [16]. (ST) system requires a better balance, quality transportation, future economic development, and environmental and social well-being [10]. (ST) can be considered a major contribution to the bigger picture of sustainability, which includes an overview of environmental, social, and economic progress, commonly referred to as the dimensions of sustainability [15].

### 2.2 Sustainable transportation: dimensions

The Brundtland report, in the definition of (SD), has mentioned three environmental, economic, and social dimensions [10]. The world bank has accepted these three environmental, economic, and social dimensions to express the concept of (SD) [17]. Subsequent reports of the (UN) have emphasized the three environmental, social, and economic dimensions of (SD) [18]. Transportation has notable effects on environmental, social, and economic dimensions and is vital in sustainability [19].

**Fig. 1:**
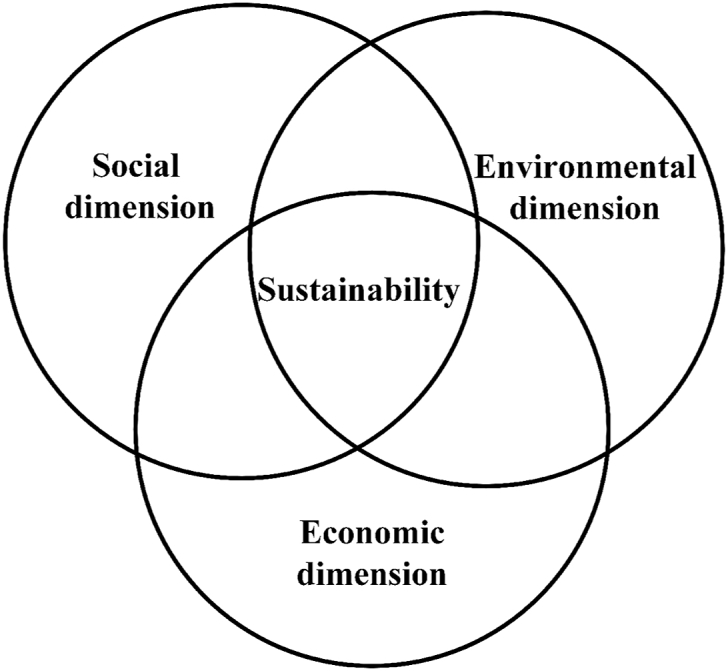
(ST) dimensions.

Several authors have introduced the dimensions of (ST) as environmental, social, and economic [2,10,15,19,20]. The other hand, finding the right balance between the conditions (present and future) in environmental, social, and economic dimensions in the concept of (ST), It has been declared important [8]. Challenges around sustainability and (ST) often emphasize that (ST) has three environmental, social, and economic dimensions, that must be considered simultaneously. If we want to show the conceptual form of importance and simultaneous attention to these three dimensions, the appropriate state can be Fig. (1).

### 2.3 Sustainable transportation: indicators

If anybody doesn't know how is sustainable or unsustainable the current transportation system, likely and exactly doesn't know what to do must about it [7]. To quantitatively measure the level of sustainability of the transportation system, we need indicators that can be measured. Indicators are variables used to quantitatively and qualitatively evaluate the sustainability of transportation projects. Of course, some of these indicators may not be applicable in some countries. Analyzing the importance and prioritization of sustainability indicators is necessary for traffic and transportation planning [19]. Sustainability indicators should be able to show and measured quantify the social, economic, and environmental effects of a transportation system [20].

Measuring the changes in indicators, their behavior, and effects in different transportation systems are quantified. The indicators partly indicate the directions the authors want (ST) to be directed. Many efforts have been made to develop (ST) indices [19]. There is no limit on the number of indicators for evaluating. A set of different indicators that reflect the different goals of transportation sustainability should be used [15]. Litman and some authors have made various attempts to list transport indicators, for example, the following, can be mentioned: proper use of energy, control of emission CO2, control of emission toxic substance and harmful, appropriate use of land, preventing the destruction of natural areas, reducing waste, upgrade safety, reducing traffic, reducing noise pollution, reducing the health consequences of transportation, reducing the cost of accidents, reducing congestion, diversifying public transportation methods, increasing Access to public transportation, balancing family costs in transportation, increasing the share of the transportation in economic well-being [8]. Indicators be selected based on eight principles: comprehensiveness, quality, comparability, comprehensibility, transparency, cost-effectiveness, and net and combined effects [20].

Litman believes that “comprehensiveness indicates environmental, social, and economic activities, and quality indicates the quality of data from reliable sources” [20]. Indicators should be transparent and cost-effective and able to provide better sustainable solutions [20]. (ST) indicators should evaluate the environmental, social, and economic effects of transportation systems by systematically examining them. Economic indicators should measure possible effects on economic well-being and macroeconomic changes such as GDP, economic efficiency, income distribution, and unemployment rate. Social indicators should reflect impacts on social and individual quality of life, such as health and safety. Environmental indicators should reflect effects on environmental qualities, such as the use of sources, (GHG) emissions and wastes, soil quality, climate, and air quality that may affect human (and non-human) life [8].

## 3 Methodology

This research has been done according to the indicators of (ST) dimensions and based on asking experts about the performance of these indicators in a factual transportation system and using the importance-performance analysis method.

In this regard articles related to definitions, dimensions, and indicators of (ST) were searched on reliable scientific sites. After extracting the widely used indicators from these articles using the verbal spectrum table, the scores of these indicators determined in terms of importance and performance in the transportation of Tehran by experts with scientific qualifications and experience in the transportation industry.

The (IPA) method was used to analyze the situation of (ST) indicators in Tehran city transportation. This method, presented by (Martilla and James, 1997), evaluates two components of importance and performance of determined indicators at concurrent. The indicators' locations were determined using the formulas of this method and a four-zone diagram. According to the characteristics of the location area of each indicator, a specific policy could propose regarding its importance and performance. The steps of this research are as follows:

### 3.1 Articles identification

#### 3.1.1 Step-1

On the websites of www.scholar.google.com and www.sciencedirect.com and www.emerald.com and www.taylorandfrancis.com, research was done by topics: “literature review” and “sustainable” and “transportation” or “transport” or “mobility” and “urban” and “pollution” and “traffic” and “safety” and “health” and “quality of life” and “metropolitan” and “definition” and “dimensions” and “indicators” and (1990–2023). In this step identified, 2895 articles. According to Brundtland's report in 1987, which emphasizes the simultaneous attention to the three environmental, social, and economic dimensions in development programs, the time frame for searching articles was determined with a short interval from 1990 onwards.

#### 3.1.2 Step-2

Most of the articles identified in step-1 were from the Google Scholar (GS) website (2520 articles). To identification of the articles more accurately, and second search was done by topics on (GS):

“Title-abstract-keywords” and “[topics step-1]”. In step-2 identified, 1020 articles on (GS).

In this way, the articles identified from four websites in two steps were 1395 articles. The complete specifications of these articles were recorded in an Excel file. Then by applying the “Remove duplicates” command in the article name column, the duplicate articles were deleted. The of articles remaining at this stage was 941.

### 3.2 Articles filtering and selecting

#### 3.2.1 Step-3

A The articles identified in steps (1–2), filtered by this question:

Is the article related to the subject and goal of the research? (Yes or no). In this step, (397 articles) were selected.

B Then, we filtered the articles (397 articles) by this question:

Are ‘definition’ or ‘indicators’ or ‘dimensions’ for (ST) provided in articles? (Yes or no). In this step, (85 articles) were selected.

**Fig. 2:**
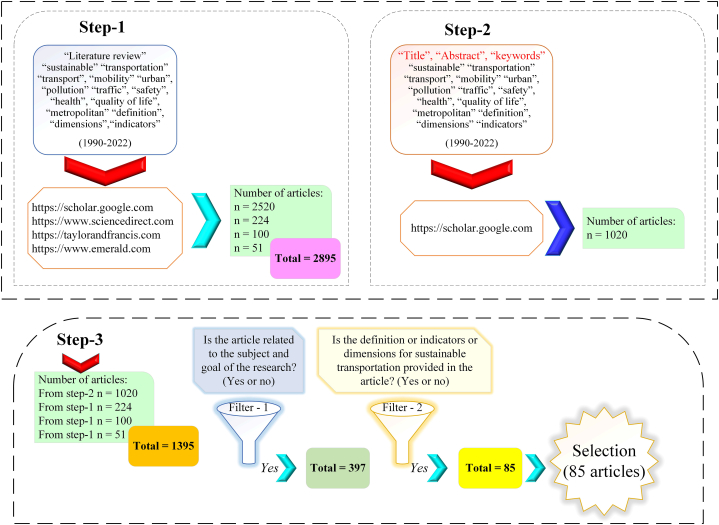
Selection articles.

The process of conducting steps (1–3) of the research is according to Fig. (2).

### 3.3 Extracting definitions and indicators

To extract the definitions and indicators of (ST), selected articles (85 articles) were analyzed. In the provided definitions, special words are highlighted and separated. The special words emphatic in the definitions of (ST) were fourteen items in all articles. Their frequency and percent were determined and recorded.

Twenty indicators were identified and recorded for each dimension (environmental, social, and economic). These indicators are mentioned in some of the 85 articles. Indicators were recorded in three separate tables and coded. The indicators coding method are:

For example: (Environmental indicator 1 = ENI1, Social indicator 1 = SOI1, Economic indicator 1 = ECI1, and etc.).

### 3.4 Analyze method

To evaluation of the performance indicators used (IPA) method. This method was presented by (Martilla and James, 1997) as an insightful, analytical, and interpretive method for simultaneously identifying the strengths and weaknesses of a system in different areas and prioritizing and finding ways to improve. Tehran is a metropolis with a population of nine million that has been plagued by negative transportation consequences. According to the website of Tehran air quality company [21], in recent years, the air in Tehran has been unhealthy on most days. Tehran city has public transportation of subway, bus, and taxi, with high average life, and more people use private cars and motorcycles.

Considering the daily air pollution of the Tehran metropolis, congestion, and high traffic, this city selected for the case study.

The table of indicators and the verbal spectrum Table (1), were sent to 20 experts. These experts had a relevant university education and 15 years of undergraduate or managerial experience in the transportation field. We asked experts to rate the importance and performance of each indicator in transportation's Tehran based on the verbal spectrum. After receiving expert answers, was created the importance-performance super-matrix of expert opinions.

**Table 1:** Verbal spectrum table for scoring the importance and performance of indicators.

Importance of indicator	Very low	Low	Middle	High	Very high
Numerical value	1	2	3	4	5
Performance of indicator	Complete opposite	Opposite	Neutral	Agree	Completely agree
Numerical value	1	2	3	4	5

### 3.5 Analyzing information

(IPA) method parameters are calculated by (1), (2), (3), (4).

(1) $$\text{The}\;\text{final}\;\text{value}\;\text{of}\;\text{importance}\;\left(\text{geometric}\;\text{mean}\right):B_{j}=\sqrt[{n}]{\prod_{j=1}^{m}b_{j_{P}}}$$

j = 1, 2 …, m &p = 1, 2 …, n

n: number of research experts.

m: number of indicators.

b_j_: final value of importance indicator.

b_jp_: the specified value for the importance of each indicator by each expert.

(2) $$\text{The}\;\text{final}\;\text{value}\;\text{of}\;\text{the}\;\mathrm{performance (geometric}\;\mathrm{mean)}\;:C_{i}=\sqrt[{n}]{\prod_{i=1}^{m}C_{i_{P}}}$$

i = 1, 2 …, m &p = 1, 2 …, n

n: number of research experts.

m: number of indicators.

c_i_: final value of performance indicator.

c_ip_: the specified value for the performance of each indicator by each expert.

(3) $$\text{Threshold}\;\text{value}\;\text{of}\;\text{the}\;\text{importance}\;\text{of}\;\text{indicators}:\mu_{b}=\frac{\sum_{j=1}^{m}b_{j}}{m}$$

(4) $$\text{Threshold}\;\text{value}\;\text{of}\;\text{performance}\;\text{indicators}:\mu_{C}=\frac{\sum_{i=1}^{m}C_{i}}{m}$$

μ_b_: threshold value of indicators importance (arithmetic mean for impotence of indicators).

μ_c_: threshold value of indicators performance (arithmetic mean for performance of indicators).

m: number of indicators.

**Fig. 3:**
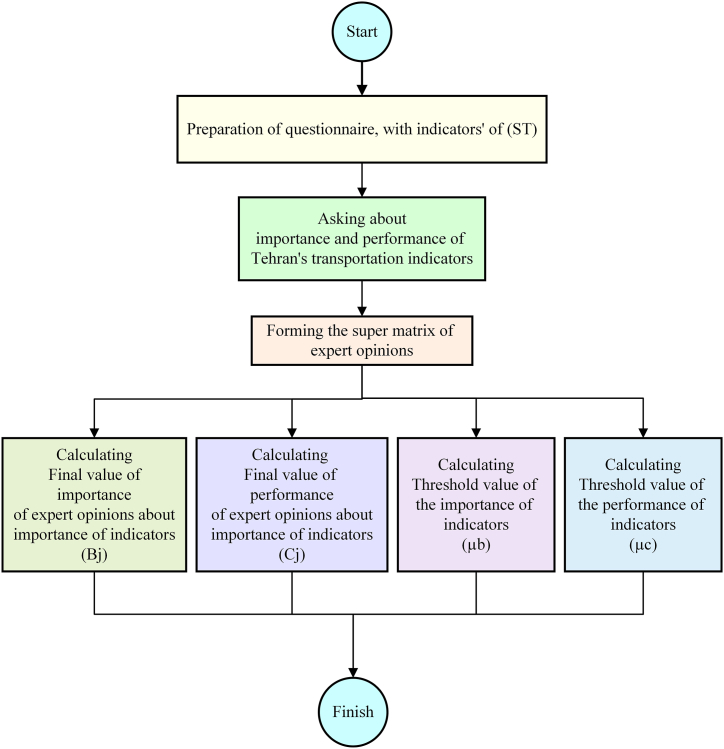
Asking opinions and analyzing information process.

The process of asking opinions and analyzing information is according to Fig. (3).

### 3.6 Determining the placement location of indicators

**Fig. 4:**
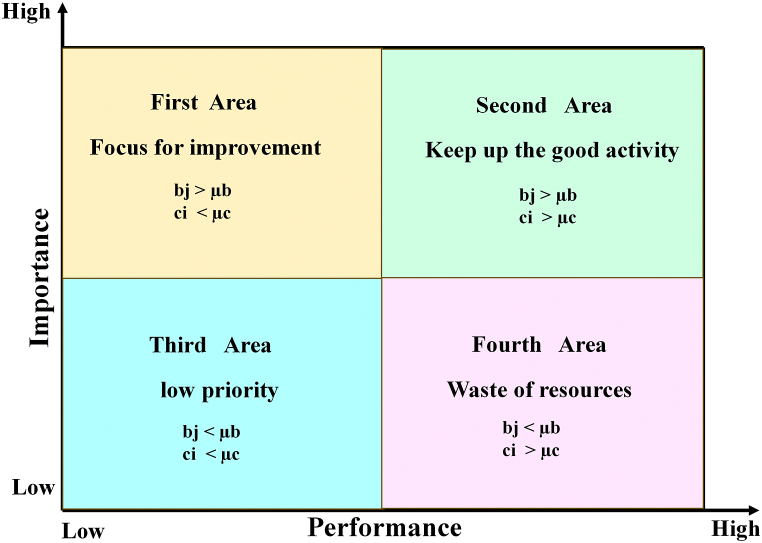
Determining location of indicators with calculations and pattern of (IPA).

Fig. (4) provided to determine the location of indicators by Martila and James. After calculations for indicators by (IPA) method, their locations are determined using the pattern of this figure.

First location: Any indicator placed in this location has high importance and low performance. The performance indicator must improve.

Second location: Any indicator placed in this location has high importance and performance. This situation must be maintained.

Third location: Any indicator placed in this location has low importance and performance. This indicator does not have any priority.

Fourth location: Any indicator placed in this location has low importance and high performance. As a result, resources have been wasted, and policies and programs must be revised.

### 3.7 Prioritizing indicators using the following relationships: (hearing voice of customer)

Prioritization of indicators helps decision-makers and policymakers to properly manage and direct resources to reach the desired result and produce satisfaction sooner. To achieve this goal, the (IPA) utilizes (5), (6) to calculate the prioritization of indicators. This approach is “hearing voice of customer” in the (IPA) method.

(5) $$ow_{j}=\left|\left(b_{j}-c_{i}\right)\cdot b_{j}\right|$$

(6) $$sw_{j}=\frac{\text{owj}}{\sum_{j=1}^{m}ow_{j}}$$

$$0\leq sw_{j}\leq 1$$

$$\sum_{j=1}^{m}sw_{j}=1$$

In the method (IPA), any indicator with higher sw_j_ has a higher priority for improvement.

## 4 Results

### 4.1 Frequency of selected articles

**Fig. 5:**
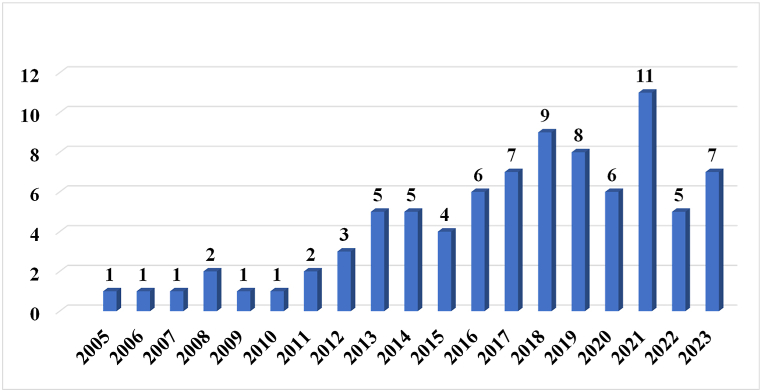
Frequency of selected articles in reviewed time period.

Fig. (5) shows the frequency of the selected articles (85 articles) in reviewed time period.

Fig. (5) shows that in the last decade, the attention of authors attention to the definition and indicators of (ST) has increased, and research in this field is on the rise.

Table (2) shows which journals published the selected 85 articles.

**Table 2:** Frequency of articles in different journals.

No.	Journal name	Frequency
1	Journal of Transport & Health	3
2	Sustainable Cities and Society	5
3	Journal of Transport Geography	4
4	Sustainable Production and Consumption	4
5	Transportation Research part E	6
6	Transportation Research Interdisciplinary Perspectives	3
7	Journal of Transport Policy	5
8	Transportation Research Part D	3
9	Transportation Research Part B	1
10	Research in Transportation Business & Management	6
11	Transportation Research Part A	4
12	Journal of Cleaner Production	13
13	Journal of Applied Energy	3
14	Journal of Cities	11
15	Research in Transportation Economics	5
16	Transportation Research part F	4
17	Journal of Heliyon	3
18	Economics of Transportation	1
19	Environmental Innovation and Societal Transitions	1
Total:	–	85

### 4.2 Widely used terms in definitions

In Table (3), fourteen widely used terms in definitions of (ST) are extracted and presented. These terms were used in definitions of (ST) in some of the 85 articles selected.

**Table 3:** Frequency widely used terms in definitions of (ST) in 85 articles.

No	widely used terms in definitions of (ST) in 85 articles	Frequency	Percent
1	Providing the needs of current generation	36	42.35
2	Keeping the ability to provide for the needs of future generations	36	42.35
3	Air pollution	24	28.24
4	Renewable	22	25.88
5	Non-renewable	22	25.88
6	Safety	19	22.35
7	Resource consumption	18	21.18
8	Emission of (GHG)	16	18.82
9	Price	16	18.82
10	Noise	15	17.65
11	Access	14	16.47
12	Health	14	16.47
13	Sexual equality	13	15.29
14	Justice	12	14.12

The results of Table (3) show that the terms, providing the needs of the current generation, keeping the ability to provide for the needs of future generations, and air pollution and renewable and non-renewable used in more than 25% of articles. Also, the results show that providing the needs of the current generation and keeping the ability to provide for the future generation's needs with 42.35% have the highest using percent in the selected articles.

### 4.3 Indicators

In Table 4, Table 5, Table 6 indicators extracted for each dimension (ST) are presented. These tables show the frequency and percentage of indicators presented in some of the 85 articles.

**Table 4:** Indicators in environmental dimension.

Indicator code	Sustainability indicators in environmental dimension in some of 85 articles	Frequency	Percent
ENI1	Reducing the growth rate of CO2 emissions	38	44.71
ENI2	Preventing the release of all types of waste	21	24.71
ENI3	Improving the livability of the environment	18	21.18
ENI4	Reducing noise pollution	27	31.76
ENI5	Reducing the emission of suspended particles	16	18.82
ENI6	Reduction of volatile organic compounds	14	16.47
ENI7	Reducing water pollution	29	34.12
ENI8	Preventing damage and loss of natural habitats	18	21.18
ENI9	Reducing air pollution	39	45.88
ENI10	Help reduce abnormal warming effect earth	27	31.76
ENI11	Reducing (GHG) emissions	47	55.29
ENI12	Minimal use of non-renewable resources	21	24.71
ENI13	Reducing the production of waste less than the absorption capacity of the planet	15	17.65
ENI14	Reuse and recycling of industrial parts	14	16.47
ENI15	Help maintain the health of the ecosystem	54	63.53
ENI16	Prevent the development of sources of pollution	23	27.06
ENI17	Minimizing the consumption of natural resources	19	22.35
ENI18	Minimizing the use of limited resources	22	25.88
ENI19	Development of environmental, solar and biotechnology industries	17	20.00
ENI20	Standardization of engines	38	44.71

**Table 5:** Indicators in social dimension.

Indicator code	Sustainability indicators in social dimension in some of 85 articles	Frequency	Percent
SOI1	Gender equality in the use of transportation facilities	17	20.00
SOI2	Improving the quality-of-service delivery	23	27.06
SOI3	Increasing convenient access to the transportation network	26	30.59
SOI4	Safety upgrade	36	42.35
SOI5	Attention to human health in transportation policies and programs	32	37.65
SOI6	Reducing congestion	38	44.71
SOI7	Reducing of traffic	41	48.24
SOI8	Reducing accidents	29	34.12
SOI9	Reducing of waste from road vehicles	16	18.82
SOI10	Improving the quality of life	34	40.00
SOI11	Increasing public satisfaction to services	24	28.24
SOI12	Coordination of transportation systems with the limitations of the disabled	14	16.47
SOI13	Increasing fairness and justice	18	21.18
SOI14	Participation of citizens in transportation decision-making	11	12.94
SOI15	Security upgrade	23	27.06
SOI16	Increasing the variety of transportation methods	22	25.88
SOI17	Promoting walking	28	32.94
SOI18	Increase shared trips	12	14.12
SOI19	Promoting cycling	25	29.41
SOI20	Coordination and integration of public transport services	21	24.71

**Table 6:** Indicators economic dimension.

Indicator code	Sustainability indicators in economic dimension in some of 85 articles	Frequency	Percent
ECI1	Reducing overall in transportation costs	33	38.82
ECI2	Reducing household expenses for transportation	22	25.88
ECI3	Helping personal businesses	27	31.76
ECI4	Reduce use of land	18	21.18
ECI5	Reducing energy consumption	29	34.12
ECI6	Supporting a dynamic economy	21	24.71
ECI7	Supporting prosperity and economic sustainability	16	18.82
ECI8	Helping the economic growth of society	32	37.65
ECI9	Increasing the cost of using a private car	18	21.18
ECI10	Limiting parking time for private cars	12	14.12
ECI11	Increasing the tax on the purchase and sale of private cars	19	22.35
ECI12	Payment of subsidies to public transport	11	12.94
ECI13	Providing a combined ticket (can be used for all types of public transportation)	17	20.00
ECI14	Directing government funds towards public transport	16	18.82
ECI15	Creation and development of low-speed suburban railway	10	11.76
ECI16	Transparency of costs and investments in transportation	21	24.71
ECI17	Reducing overall transportation costs (vehicles, parking, road tolls and transportation services).	18	21.18
ECI18	Reducing costs of time spent in traffic	22	25.88
ECI19	Improving the quality of transportation for disadvantaged people	26	30.59
ECI20	Reducing transportation costs for the government (annually, per GDP)	14	16.47

Table (6) shows the frequency and percentage of 20 indicators widely used in the economic dimension among the 85 selected articles. Among the 20 economic indicators, the indicators of ECI1, ECI3, ECI5, ECI8, and ECI19 (25%) in more than 30% of the articles, have been attention on by the authors. The results of tables (5), (6), and (7) show which indicators have high important in (ST) for the authors of these articles.

Table (4) shows the frequency and percentage of 20 indicators widely used in the environmental dimension among the 85 selected articles. Among the 20 environmental indicators, the indicators of ENI1, ENI4, ENI7, ENI9, ENI10, ENI11, ENI15, and ENI20 (40%) in more than 30% of the articles have been noticed and emphasized by the authors.

Table (5) shows the frequency and percentage of 20 indicators widely used in the social dimension among the 85 selected articles. Among the 20 social indicators, the indicators of SOI3, SOI4, SOI5, SOI6, SOI7, SOI8, SOI10, SOI17, and SOI19 (45%) in more than 30% of the articles have been noticed and emphasized by the authors.

### 4.4 Results of the (IPA) for transportation in Tehran

By using the indicators of Tables (4–6) and the super-matrix of points given by experts, the (IPA) was carried out regarding transportation in Tehran. The results of the analysis and summary of calculations are presented in Table 7, Table 8, Table 9.

Table (7) shows the location of environmental indicators and the prioritization of indicators for improvement. This prioritization shows that indicators ENI17, ENI11, ENI10, ENI18, and ENI13 are in order of priority (1–5) for improvement.

**Table 7:**
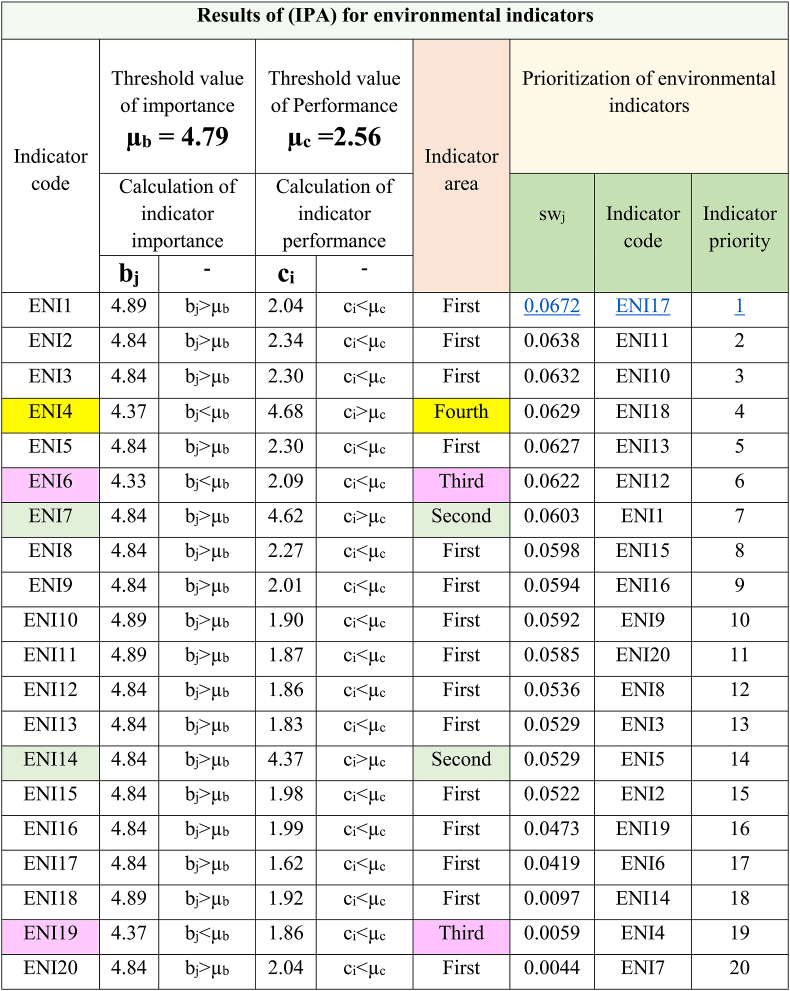
Results of (IPA) for environmental indicators.

Also, the results of Table (7) show the location of the fifteen indicators (75%) in the first area, two indicators (10%) in the second area, and two indicators (10%) in the third area. Finally, the location of one indicator (5%) is in the fourth area.

In this table the indicator “Minimizing the consumption of natural resources” with sw_j_ = 0.0672 has the first priority for improvement.

Table (8) shows the location of social indicators and the prioritization of indicators for improvement. This prioritization shows that indicators SOI5, SOI12, SOI14, SOI19, and SOI11 are in order of priority (1–5) for improvement.

**Table 8:**
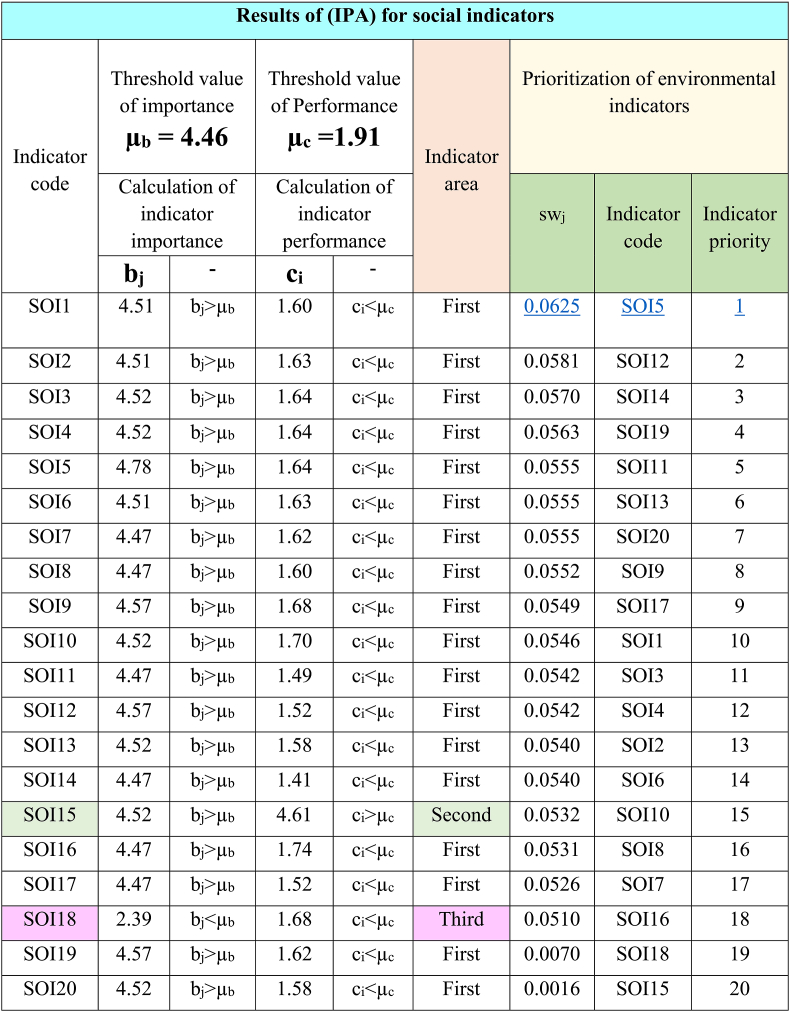
Results of (IPA) for social indicators.

Also, the results of table (8) show the location of the eighteen indicators (90%) in the first area, one indicator (5%) in the second area, and one indicator (5%) in the third area. Finally, there isn't any indicator in the fourth area.

In this table, the indicator “Attention to human health in transportation policies and programs” with sw_j_ = 0.0625 has the first priority for improvement.

Table (9) shows the location of economic indicators and the prioritization of indicators for improvement. This prioritization shows that indicators ECOI5, ECOI8, ECOI19, ECOI6, and ECOI7 are in order of priority (1–5) for improvement.

**Table 9:**
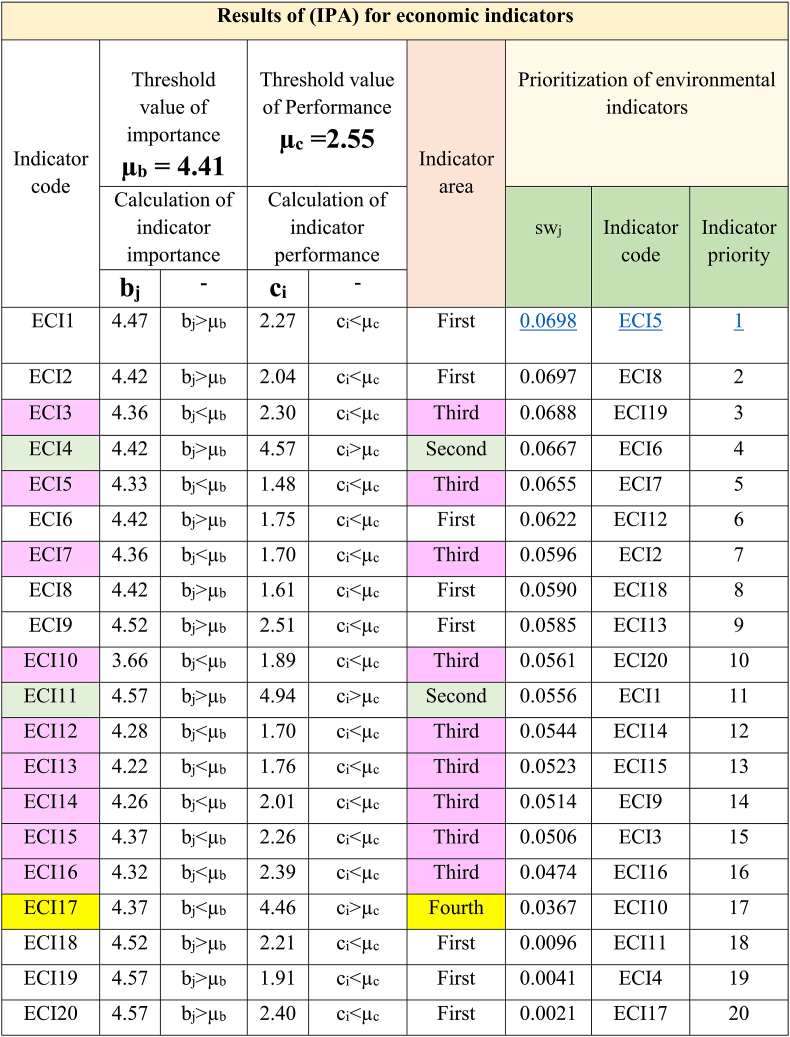
Results of (IPA) for economic indicators.

Also, the results of Table (9) show the location of the eight indicators (40%) in the first area, two indicators (10%) in the second area, and nine indicators (45%) in the third area. Finally, the location of one indicator (5%) is in the fourth area.

In this table, the indicator “Reducing energy consumption” with sw_j_ = 0.0698 has the first priority for improvement.

### 4.5 Determining location of indicators of (ST) in Tehran's transportation

Based on the results of Table 7, Table 8, Table 9, and by pattern Fig. (4), the location and frequency of the indicators of transportation dimensions of Tehran city have been determined.

**Fig. 6:**
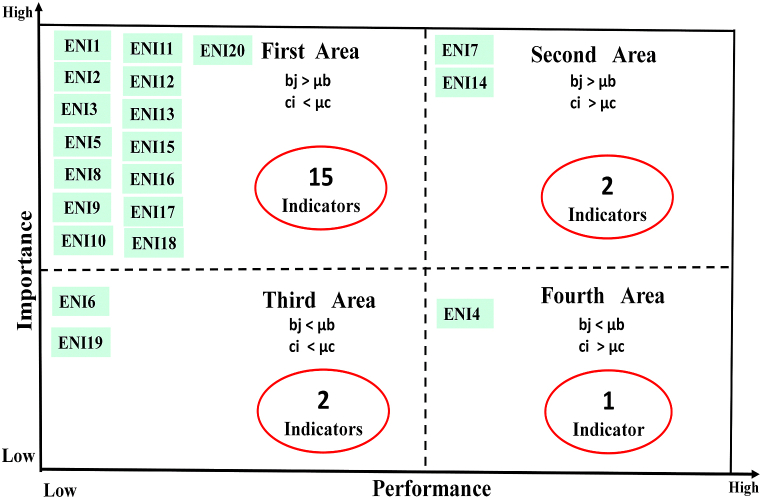
Location and frequency of environmental indicators.

According to Fig. (6), 75% of the indicators of the environmental dimension (fifteen items) are in the first location. These indicators have high importance and low performance. The performance of these indicators needs to be improved. 10% of indicators (two items) are in the second location. These indicators have high importance and performance. This condition needs to be maintained. 10% of indicators (two indicators) are in the third location. These indicators have low importance and performance, and these indicators do not have priority. One indicator (5% of indicators) is in the fourth location. This indicator has low importance and high performance. Focusing on the continued performance of this indicator causes a waste of resources.

**Fig. 7:**
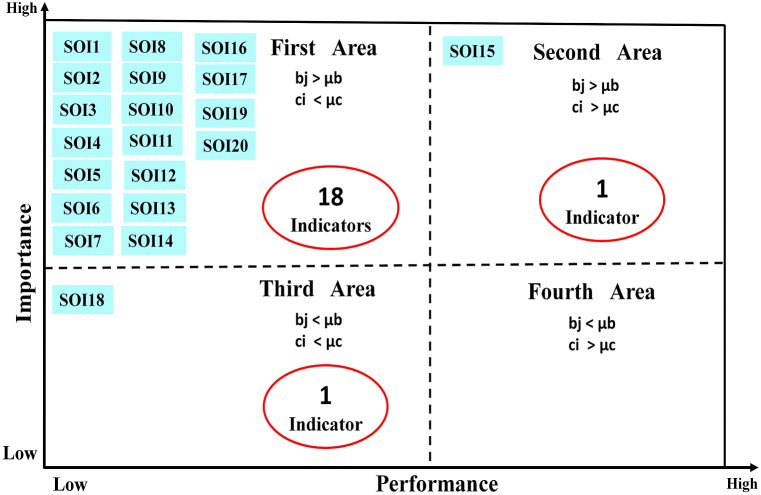
Location and frequency of social indicators.

According to Fig. (7), 90% of the indicators of social dimension (eighteen items) are in the first location. These indicators have high importance and low performance. The performance of these indicators needs to be improved. One indicator (5%) is in the second location. This indicator has high importance and performance. This condition needs to be maintained. One indicator (5%) is in the third location. This indicator has low importance and performance and doesn't have any priority.

**Fig. 8:**
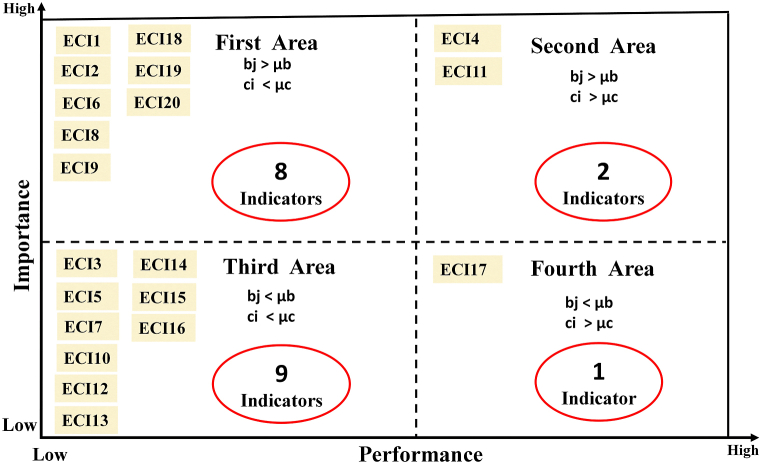
Location and frequency of economic indicators.

According to Fig. (8), 40% of the indicators of economic dimension (eight items) are in the first location. These indicators have high importance and low performance. The performance of these indicators needs to be improved. 10% of indicators (two items) are in the second location. These indicators have high importance and performance. This condition needs to be maintained. 45% of indicators (nine indicators) are in the third location. These indicators have low importance and performance, and these indicators do not have priority. 5% of indicators (one indicator) are in the fourth location. This indicator has low importance and high performance. Focusing on the continued performance of this indicator causes a waste of resources.

## 5 Discussion and recommendations

The information presented in Table (3) indicates the current standing of special terms utilized to define (ST) in 85 articles. A total of fourteen widely used special terms have been identified. To fulfill the first part of the goal A this research, based on the findings in Table (3), we have presented a comprehensive definition of (ST) which includes all fourteen of these widely used special terms. Our proposed definition for (ST) is:

“(ST) provides by saving and optimally using natural resources and keeping the ability to provide the needs of future generations, the needs of the current generation. Uses renewable resources less than the production rate and uses non-renewable resources less than the rate of development of renewable alternatives. It controls the emission of (GHG) to prevent abnormal global warming and helps the health of communities by limiting air pollution and reducing noise pollution. (ST) prioritizes social justice and gender equality for users by providing affordable and accessible services and high safety and convenience.

Also, in response to part A of the objectives of this research, among the 85 selected articles, 60 widely used indicators in the environmental, social, and economic dimensions of (ST) have been extracted and presented in Table 6, Table 7, Table 8.

To achieve another goal of this research, the (IPA) method was used to evaluate and prioritize transportation indicators in Tehran. The results of Table 7, Table 8, Table 9 clearly show the priority of improving performance of indicators. Respectively, the first priority between indicators for the environmental, social, and economic dimensions are “Minimizing the consumption of natural resources” (sw_j_ = 0.0672), “Attention to human health in transportation policies and programs” (sw_j_ = 0.0625), and “Reducing energy consumption” (sw_j_ = 0.0698). Subsequent indicators are of lower priority. These findings highlight the significant challenges of Tehran's transportation system, including excessive energy usage, depletion of natural resources, and threats to human health.

In order to make the research findings easier to understand, Table (10) was created using data from Fig. 6, Fig. 7, Fig. 8.

**Table 10:**
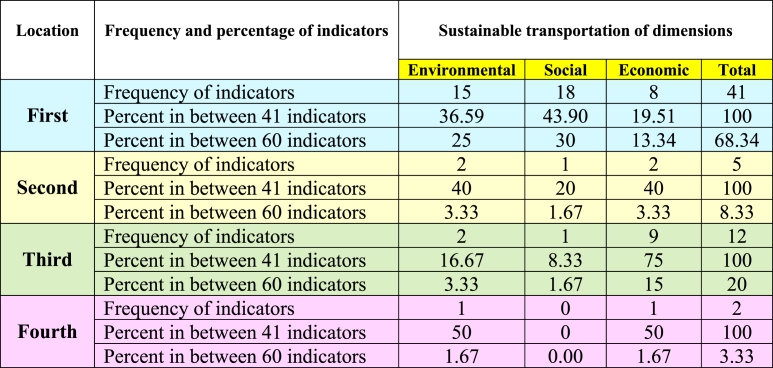
Frequency and percentage of indicators in various locations.

Table (10) shows that out of 60 indicators evaluated in the three dimensions of sustainability, 41 indicators (68.34%) are in the first location. Most of these indicators are related to the environmental and social dimension, which is directly related to the quality of life and satisfaction of the residents of Tehran. According to the information in Table (10), it is evident that the experts are respectively most worried about the social, environmental, and economic indicators and their impact on the quality of transportation in Tehran. The 41 environmental and social indicators have high importance. However, their performance is low. It is necessary to change transportation policies to improve the performance of these indicators. Most of the negative consequences of transportation are given rise to low performance in environmental and social indicators. Some of the harmful effects of environmental indicators are: consumption of fossil fuels, excessive use of land, destruction of natural habitats, pollution of air, water, and soil, sudden changes in weather, global warming, melting of polar ice, and weather inversion in big cities. Each of these consequences directly affects the quality of life and general health of humans. Also, the negative consequences of the low performance of social indicators can cause people's dissatisfaction, injustice, or decrease in social justice, increase in poverty, decrease in the level of welfare of low-income groups, decrease in safety, and increase in traffic.

Based on the summary of information in Table (10), five indicators (8.33%) were located in the second location. These indicators hold significant importance and are performing well. It is imperative to maintain this favorable situation. Also, the information in Table (10) shows that twelve indicators (20%) are in the third location, and the importance and performance of these indicators are low. According to expert opinions, these indicators didn't have priority and can ignored.

Eventually, the information in Table (10) shows that two indicators (3.33%) are in the fourth location. These indicators have low importance, but their performance is high. Despite the low importance, to have high performance in these indicators is essentially a wasteful use of resources, and it's necessary to revise policies.

According to the above, our recommendations for improving Tehran's transportation situation in three environmental, social and economic dimensions are:

To improve Tehran's transportation system is needed, a comprehensive approach. That includes improving the quality of consumed fuel and electrifying city buses to reduce emissions and promote sustainability. Renovating and improving public vehicles will enhance their functionality and provide a more pleasant commuting experience, all for citizens and passengers. Supporting health-oriented transportation such as cycling and walking is crucial, and this can be achieved by improving cycling and walking paths throughout the city. Developing the subway and suburban trains will further expand the public transportation options available to residents.

In order to boost the usage of public transportation, it would be beneficial for the government to allocate aid towards supporting these systems and give subsidies to students, government employees, and those who are vulnerable or have low incomes. To guarantee safety and efficiency it's important to impose rigorous standards and conduct technical inspections for private and public vehicles. Organizing passenger stations and developing specific routes and express services for public transportation would simplify the system and make it more accessible for everyone.

One way to promote the utilization of public transportation and reduce traffic congestion is to make it more expensive to use private cars by increasing parking fees, taxes on purchase and maintenance, and fuel prices. Additionally, encouraging shared trips among personal car users can also be beneficial in reducing traffic issues.

The study and research to feasibility of procedures of accessing a universally acceptable definition of (ST) and its indicators can be affected in raising the quality of the performance of transportation systems and making it easy to evaluate the adherence of countries to the requirements of (ST).

## 6 Conclusions

Transportation is undoubtedly a critical factor in the economic development of nations. However, there are numerous challenges associated with it. Despite many attempts to present a universal definition and indicators of (ST), a global agreement has yet to be reached. Without a Universally agreed-upon definition, evaluating transportation performance in different countries and determining adherence to (ST) requirements and indicators is challenging. We extracted definitions from selected articles and used 14 widely used terms to define (ST). Also, we presented a set of 60 widely used indicators in three dimensions (ST), and using these indicators and the (IPA) method, we evaluated the importance and performance of these indicators in Tehran's transportation.

This evaluation showed that although 41 indicators (68.34 present) have high importance in Tehran transportation, their performance is low and needs to be improved. Also, this evaluation showed that the experts' concern about the environmental and social consequences of Tehran's transportation is more than its economic consequences. The change in Tehran's transportation policies should be able to improve the performance of environmental and social indicators to improve the quality of life, human health, and public satisfaction. This research showed that minimizing the consumption of natural resources, paying attention to human health, and reducing energy consumption have high priority for improvement. The main advantage of this research is the ability to simultaneously evaluate the importance and performance of indicators and prioritize them to improve performance with optimal cost and time. Using to this method is easily and quickly available in all cities and transportation systems.

However, to some limitations should be considered. These restrictions include:

1 The Variety of definitions (ST) and indicators are extensive. This variety and breadth create significant challenges in choosing a superior definition and indicators related to (ST).
2 The next challenge is to determine which indicators are most important to measure the importance and performance of a transportation system. We overcome these limitations by utilizing widely used indicators.
3 The next challenge is the dependence of the accuracy and validity of the results on the scientific qualifications and experience of experts in transportation systems. These experts must have sufficient knowledge of the transportation system in the study area.

## Ethics statement

The authors of this article declare their commitment and adherence to all the provisions of the ethical statement Journal of Heliyon. We want to make it clear that we didn't use human or animal samples in our research, so we didn't have required a special permit. Also, the share of contribution of the authors in conducting this research is equal, and the authors announced that the Journal of Heliyon has permission to publish this article.

## Data availability statement

Data included in article/supplementary material/referenced in article.

Reference: source [22]: https://github.com/isaheidari/super-matrix.

## CRediT authorship contribution statement

**Isa Heidari:** Writing – review & editing, Writing – original draft, Visualization, Validation, Software, Resources, Project administration, Methodology, Formal analysis, Data curation, Conceptualization. **Abbas Toloie Eshlaghy:** Validation, Supervision. **Seyyed Mohammad Seyyed Hoseini:** Supervision.

## Declaration of competing interest

The authors declare that they have no known competing financial interests or personal relationships that could have appeared to influence the work reported in this paper.
